# Cellular and molecular representations: Following the lesson of David Goodsell

**DOI:** 10.1002/pro.70616

**Published:** 2026-05-25

**Authors:** Monica Zoppè, Vera Celia Julia Williams, Ariane Briegel

**Affiliations:** ^1^ Institute of BioPhysics IBF‐CNR Milan Italy; ^2^ Integrative Structural Cell Biology, Department of Structural Biology and Chemistry Institut Pasteur, Université Paris‐Cité, CNRS UMR 3528 Paris France

**Keywords:** cell biology, David Goodsell, image theory, imagination, mesosphere, representation, structural biology

## Abstract

David Goodsell's images have helped, over the last 30 years, thousands of students all over the world to imagine the cellular world by representing molecules, proteins, complexes, viruses, bacteria, and cellular processes. His contributions to the development of Molecular Graphics (MG) have been invaluable, and his retirement is the last (for now) of his kind acts: opening space for the next generation of scholars in the art and science of MG. Compared to the ‘90s, we have a much wider range of objects, places, processes and information that can serve as the basis for our images, and we also have many more tools for producing them, as demonstrated by the great work of thousands of scientist‐artists, active as amateurs, as professional illustrators, or as members of dedicated institutional sections. Three decades of experience have produced a creative chaos that needs an organization effort, if it is to become “common background knowledge” not just for professionals of cellular and molecular biology, but for the wider scientific community.

## 1 DAVID'S WORK

For anyone who ever considered the appearance of proteins, the image in their mind is likely derived from David's work. His watercolor depictions feature not only on the PDB‐101 (Zardecki et al., 2022) page—the entry point for most biology students—but also in textbooks, magazines, art galleries and countless visualizations, as he generously grants permission to reuse them in other settings.

He is able to portray single proteins, complex assemblies of tens of subunits (such as the ATPase), and even large entities, such as viruses or cellular sections, conveying both their beauty and their intricacy with a perfect balance of clarity and scientific rigor. His style (Goodsell et al., 2019), of black atomic contours filled with watercolor, has become iconic, and it is unsurprising that many programs developed for protein visualization include a specific “Goodsell style” option: VMD (Humphrey et al., 1996), MolNodes for Blender (Johnston, 2025) and Mol* (Chareshneu et al., 2023) are just some well‐known examples.

I was fortunate to meet him when I had recently engaged in studies of molecular and cellular representation, at the Gordon Conferences on Visualization in Science and Education (GRC, 2007) in 2007. That encounter made me appreciate, besides his talent, also his kindness and generosity.

More recently, in 2022, I was interested in the use of colors in cellular representations, and David was one of the first persons to interview. After answering all of my questions, he added: “Let me know if you need any illustrations—all the ones I've done for the RCSB PDB are free for use.”

Once again, he was thoughtful and generous.

My interest arose while reasoning on the current state and possible directions of representations not only of proteins, but also of their environment—the many different places within cells, the so‐called mesosphere. The issue is becoming increasingly relevant; on one side, Computer Graphics (CG) continue to advance, driven by ever more powerful tools; on the other side, new scientific approaches like cryo‐electron tomography are now enabling the structural characterization of proteins in their cellular context. In particular, I had noticed that as more people produce visualizations of cells and their details, the lack of any standard for depicting specific features is making it increasingly difficult to interpret these images (Zoppè, 2022).

## 2 REPRESENTATIONS OF THE REAL WORLD

To explore the issue of visual representation of cellular biology, we can draw a parallel between the cellular world and the world that we see and perceive through our senses, henceforth referred to as the “real world” as opposed to the “cellular world.”

A first consideration is that any representation, be it in words, paintings, photographs, videos and up to virtual reality (VR), is a human construct. As such, it reflects both the depicted reality and the intent of its human authors, who emphasize what they consider most important for the viewer to see or to imagine. This is also true for verbal descriptions, testified by countless literary works spanning millennia. For the viewer, or reader, it is often easy to relate the representations to the real world, as the images typically refer to something that we already know.

**FIGURE 1:**
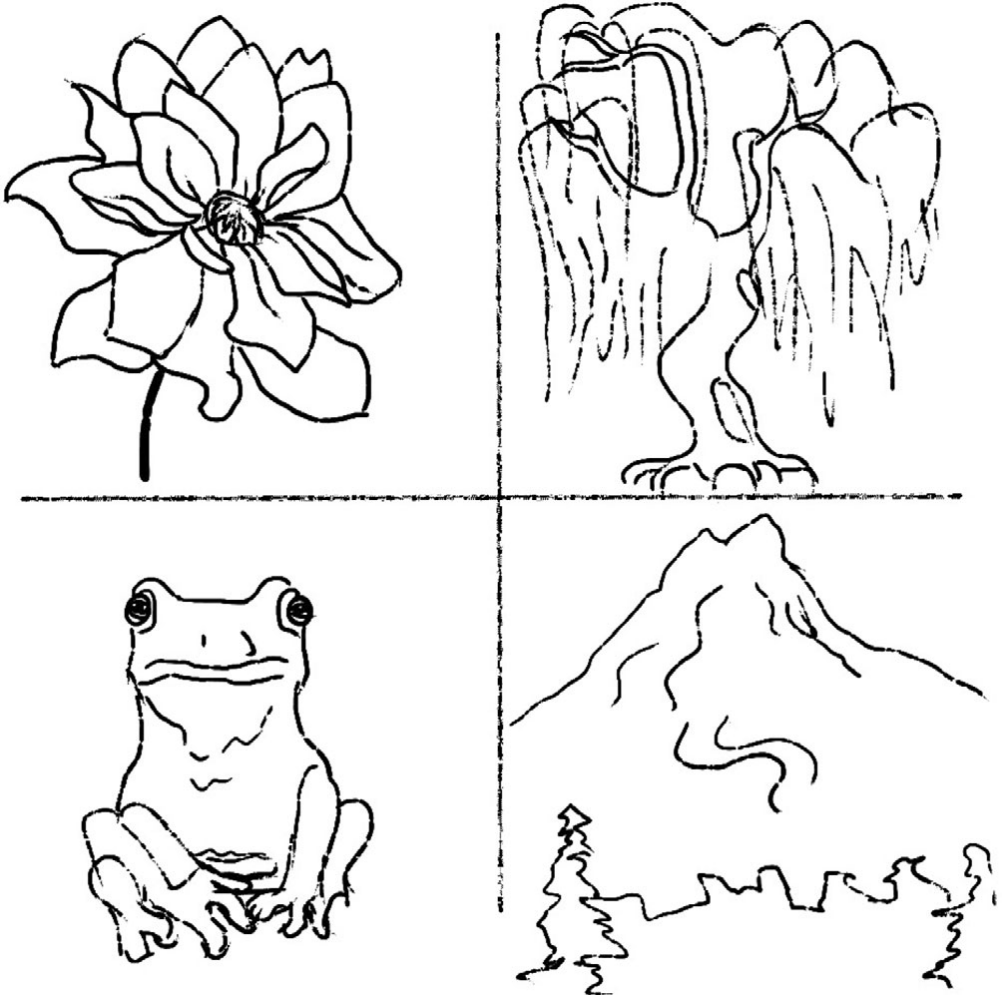
Our knowledge of the real world allows us to make sense of images even when they are just “Summaries” of the real thing. Even without considering faces, which are a special case, a simple sketch is easily recognized: flowers, trees, frogs, and mountains are all directly connected in our minds to the real things.

Frequently, a few words or a minor detail are sufficient to evoke the mental image that each one of us has in mind (see Figure 1). A single image of a gondola in a canal reminds us of Venice, just as the skyline of New York or the tail of a cat evoke the Big Apple or our favorite feline. We can imagine Venice, New York, or a cat because we have already encountered (in person or through images) the scenes and therefore possess a mental image of them. As it happens, there can be thousands of different images representing the same place/fact/object, and each one will also tell us something about the authors of the representation and their intent: the light, the framing, the angle, the moment of the day and the position of the cat's tail are authors' choices aimed at evoking different memories, sensations, and emotions in viewers. Even images of the very same place, taken with the same camera and same settings, in different seasons, or at different times of the day, will provide different sensations in viewers while the subject is always the same.

We directly and easily associate the view of a coastline lit by a thunderstorm showing a stormy sea with danger and fear. A picture of the same view, on a summer day, with a white beach and calm water, instills a sense of relaxation, vacation, and easy life.

While images can evoke memories and sensations, our knowledge of the real world is mediated by experiences that are not limited to sight: we feel the temperature, we taste food, we touch hard and soft surfaces, we hear the sound of wind and of animals, and so forth. Table 1 reports just a few examples of how our senses contribute to our knowledge of the real world.

**TABLE 1:** What we know about our world, and how: Few examples.

	Dimensions/scale	Perception
Objects	Minute splinter/mountain	Sight, touch
Environment		
Space, distances	Under blankets/middle of ocean	Sight
Light	Brightest day/darkest night (cave)	Sight (adaptation)
Temperature	Freezing cold/burning hot	Touch, inner feeling
Humidity/water	Desert dry/thickest fog, rain, water	Skin, sight
Activities	Eye gaze/flower blooming/thunderstorm	Sight, sound, smell, effects
Forces		
Wind	Gentle breeze/hurricane	Feeling, effects
Gravity	(constant)	Effect
Time	Split second/years	Inner feeling

*Note*: In the effort of representing the cellular world, the challenge is to evoke in viewers analogous feelings, connected to the forces and events that happen in the cell: for example, changes in pH, metabolic activation, Calcium waves, microtubule disaggregation and rearrangement.

Objects are the most easily represented items, but our world is not only made of tangible things and, most important, is constantly changing under the effect of many inputs, from our acts to the effects of gravity. The forces that make things happen can be intrinsically invisible, yet we do know them very well—wind, changes in temperature, gravity, and more—because we see or feel such forces and their *effects* on the world and on ourselves. We are aware of gravity because everything tends to move toward the ground; we recognize heat and cold and properly react; and we see the effects of the wind when it blows out a candle or destroys our homes. We do have means of describing in scientific, physical terms what happens, but even if we did not, gravity, wind, and heat would feel the same for each one of us. The table above also lists the range and limits of our sensitivity for the listed items.

**FIGURE 2:**
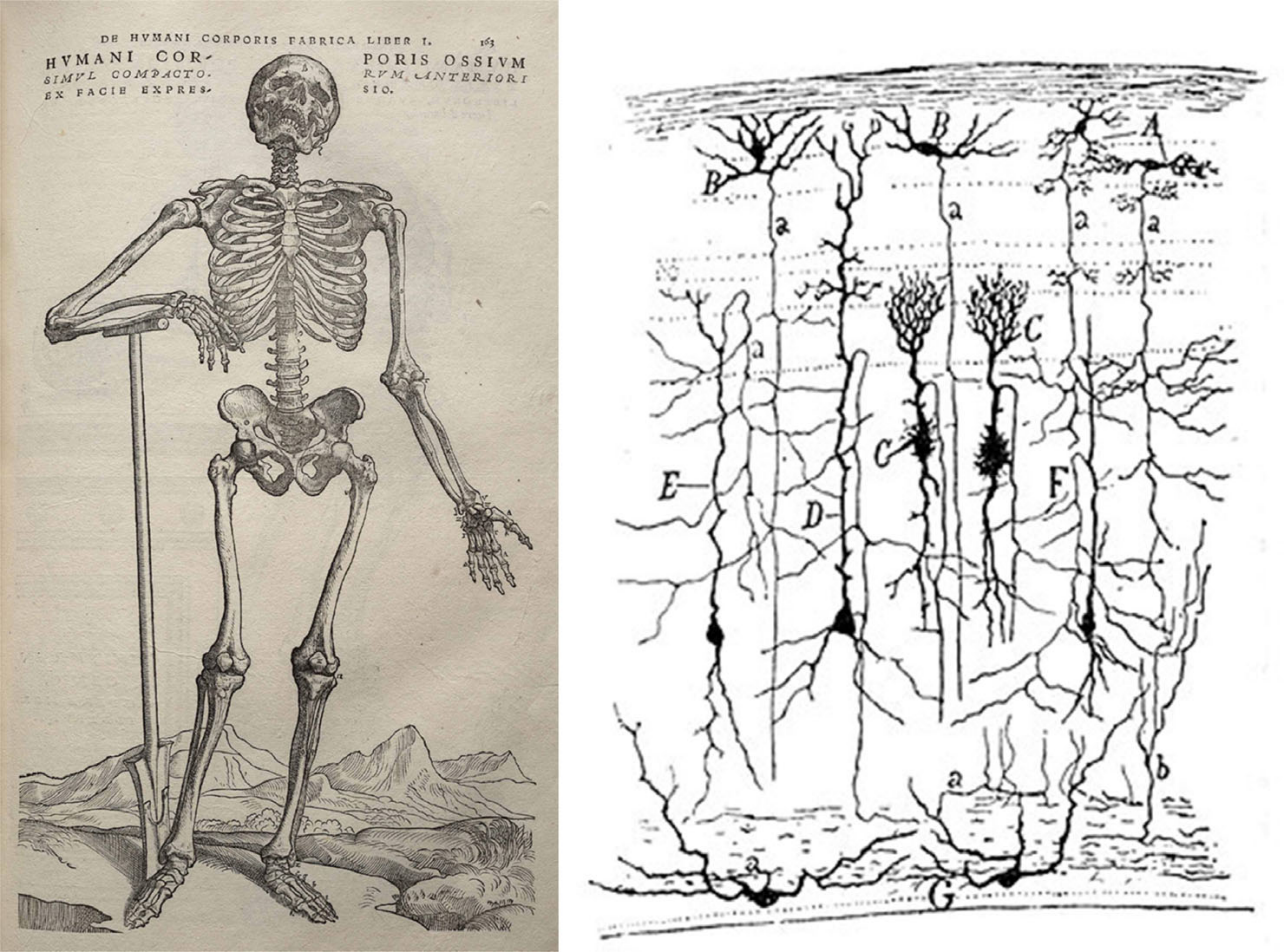
(Left) In an early example of scientific representation, Vesalius conjugated art and science, and informed viewers by removing selectively the layers occluding sight: skin, muscles, veins and arteries, and all other organs, to leave only bones and (some) cartilage. Image from Public Domain, https://commons.wikimedia.org/w/index.php?curid=458936. (Right) Ramon y Cajal extracted clear information from microscope sections: here is the drawing of a section through the optic tectum of a sparrow, from “Estructura de los centros nerviosos de las aves,” Madrid, 1905 Public Domain, https://commons.wikimedia.org/w/index.php?curid=612561.

For the description of our world one important aspect is the issue of dimensions. The span of dimensions available to our senses is wide in relation to us: we unconsciously and instantly adapt our sight from the small prints of a newspaper to the profile of a mountain in the distance. Even though it spans 7 or 8 orders of magnitude, from fractions of mm (10^−4^ m) to tens of km (10^4^ m), with everything in between, this range of dimensions is limited. Similar limitations apply to sound, touch etc., as indicated in Table 1. In addition, representations of the real world, especially in the scientific realm, go well beyond what we can directly see. We extract general principles from many observations, and thus define, for example, the parts of flowers in a systematic way; there are many beautiful representations of categorical aspects of life, as masterly exemplified by the work of Vesalius, who described the inner working of the human body by categories (bones, muscles, circulatory apparatus etc.), in a great example of how art can meet science and deliver representations that are at the same time beautiful, scientifically accurate, and very informative (Figure 2, left). As a step in between the visible and the nanoscopic, we can consider the work of Ramon y Cajal; through an operation of “induction of visibility,” (Fiorentini, 2011) he could extract information from microscope slides of brain, infer the position and organization of neurons, and make them visible as clear drawings (see Figure 2, right).

In short: we know through direct experience the world that we live in; we can represent it in many forms, and we can interpret the representations by referring to what we already know.

## 3 REPRESENTATIONS OF THE CELLULAR WORLD

Some caveats to start with:

- There is no typical cell: each one, from the simplest to the most complex, is different. We refer to *the cell* as we generalize features that, although in different forms, belong to most cells. Some major distinctions are made only for prokaryotic, plant, and animal cells.
- Cells do not live in a vacuum: they are surrounded by other cells, or at least by a watery medium.
- We do know many types of cells, but undoubtedly there are many that we do not yet know.

Cells are way too small to be explored directly with our senses, but fortunately, we do have a wide range of information that allow us to describe them. Some of this information derives from data which are collected through scientific instruments and made accessible to us in a visual form. Even drawings from the early light microscopes, in which the images were collected (as still are) directly by the eye of the observers as in the case of Ramon y Cajal, are by definition distorted by the effect of lenses. More modern light microscopes, which use combinations of lenses and selective light sources, coupled with interventions on the observed cellular features and with sophisticated data management, provide information that is more and more processed. Of course, we take any possible measures to make sure that what we finally see has a strong correspondence with the “ground truth” of the cellular world, but the undeniable fact is that our descriptions of cells are necessarily mediated by a growing number of inferences as the observed objects get smaller and smaller. We can therefore conclude that whatever we think we see is always a representation, that is, as we mentioned before, a human product, made with the aim of showing to the viewer what the human maker wants the viewer (often her/himself) to see. Does this mean that such images of cells are “false”? Certainly not: they are “representations,” just like Vesalius paintings of veins and muscles.

The problem with representations of cells is that we do not have direct experience of the cellular world. In order to get a “realistic perception” of life in the cell, a possible approach is to *imagine* what we could see and experience if we were able to “visit” a cell as we normally visit our world.

As a guiding analogy for such visit, we can think of a cell as if it were a town, as discussed previously in Zoppè (2017). We all have experience of visiting towns and cities, and we know several facts of urban life even if we do not see them. For example, we all know about general features and infrastructures of places: some are visible (e.g., roads and buildings), some are hidden (e.g., water pipes and electric lines). Cities are filled with people and other animals of many different sizes, trees and flowers, gardens and balcony pots.

Like cells, cities never sleep: there are moments of high and low activity, cycles of day and night, seasons, sunny and rainy days, and sometimes exceptional events. When visiting a new place, we organize our itinerary according to our time and interests. Well before we enter the place we already have a mental image of what to expect: we may have seen photographs, maps, films and descriptions from earlier travelers. Many activities occur inside buildings: factories, schools, homes, and so forth, invisible to us unless we go inside.

If we adopt a *tourist attitude*, trying to imagine a cell like a town (ignoring for the moment the fact that cells develop in full 3 dimensions, while cities are mostly planar with limited excursion on the vertical axis), we can try to organize a visit and to build our mental image of what we might see. This journey is not meant to explain, not even to understand nor to describe the cell in a scientific sense. We do it for our own enjoyment, eager to see and admire the new place, the complexity, the organization, and the harmonious dance of activities that surround us when we are in nature. Then, each person has different preferences and ways of looking, and will be attracted by different aspects of the place, just like architects and historians or sociologists approach cities differently. The places do not change: it is our perspective that does.

The task of imagining, or building a mental image of a cell, is greatly facilitated by transforming everything to dimensions that we can easily perceive: Table 2 reports some cellular objects, as seen in the Perceptive Scale mentioned above (Zoppè, 2017): by multiplying the size of the cellular world by ten million times, this scale allows us to imagine its objects, features and activities in direct relation to us. We can enter organelles as if they were buildings, see the cytoskeleton as made of ropes or pipes, and take up proteins or protein complexes as if they were animals, from frogs to cows.

**TABLE 2:** The cell at perceptive scale of ten million times.

Cell	5–50 μm	Village, small lake	50–500 m
Internal structures			
Nucleus	3–15 μm	Sports field, big (10 floor) building	30–150 m
Golgi	1–5 μm	Medium building (3–6 floor), airplane	10–50 m
Membrane (thickness)	6–8 nm	Wall (internal); front door	6–8 cm
Ribosome	30 nm	Cat	30 cm
Proteins			
GFP, Actin	3–4 nm	Apricot	3–4 cm
Spectrin	100 nm	Snake	1 m
NFκB complex	12–12 nm	Grapefruit	10–12 cm
DNA			
DNA double helix	2 nm	Small pipe (garden hose)	2 cm
DNA human genome	2 m	Half Earth circumference	20000 km
Other molecules			
ATP	1.5 nm	Cherry	1.5 cm
Ca++ ion	0.2 nm	Flea	2 mm
Ca++ ion (hydrated)	1.2 nm	Hazelnut	1.2 cm
Water	0.28 nm	Small ant	2.8 mm
Sugar (glucose)	0.6 nm	Pea	6 mm
Cholesterol	2 nm	Bee	2 cm
Aminoacids (Gly‐Trp)	0.6–1 nm	Poppy seeds	0.6–1 cm
Virus (HIV)	100 nm	5–6 years old human	1 m

*Note*: Some cellular objects would have perceivable sizes, if we think of them as amplified by ten million times.

Cellular images have been produced for many years, at many levels of detail, and with increasing resolution. Conceptual representations have been printed in journals and textbooks: somewhat simplified, sometimes stereotyped. There are accepted representations of the major cellular elements that any biologist will recognize. As the system gets populated, it becomes possible to put things together and virtually build a “digital cell” in visual terms.

Still, there are “things” that are not normally represented, but are part of the picture, especially the dynamics and the forces that drive them. The wind moves the leaves on trees; we see the shaking leaves and recognize the wind. Calcium ions make calmodulin open: can we detect the calcium wave? Can we transmit to viewers the information that something is happening?

We need to use our creativity to build images that will describe what we know. Scientists, biologists and scientific illustrators who are familiar with these concepts have used colors and other signs to convey each specific information: the problem is that on one side everyone uses their own aesthetic and cultural reference (hence the need for a legend in each image), and on the other side the concepts themselves are unknown to everyone except scientists. If we can *show* in consistent ways the various forces, even people with no knowledge of their physical nature should become able to recognize the clues and their effects when they meet them.

Readers will have noticed that there is an element of circularity in this imagination effort: without direct experience of the cell, we have to build our mental images not on the real things, but on their representations.

Much of our visual knowledge of cells comes from two sources: optical microscopy and molecular structures, at the two ends of the dimension spectrum. The visual space in between, the mesosphere, is getting defined better and better every day, thanks to recent advances in imaging techniques such as cryo‐electron tomography and to improvements in image processing and visualization. Building on these sources, several groups and individuals have attempted a reconstruction of cellular landscapes. David's paintings are among the best known, and they have inspired many others: for example work from his former colleagues at Scripps (see, e.g., Johnson et al., 2015; Maritan et al., 2022) and from other groups (Berry, n.d.) including our (SciVis, n.d.), and several commercial ones (see, e.g., Johann Steinegger, n.d.; XVIVO, n.d.). The most recent visualizations are masterpieces of Art and Science, and there are also very interesting attempts at providing interactivity in the cell medium, playing as a cat inside an algal cell (Chen, 2023; Steam, n.d.), or invisibly inside a fungal cell (Johann Steinegger, n.d.).

**FIGURE 3:**
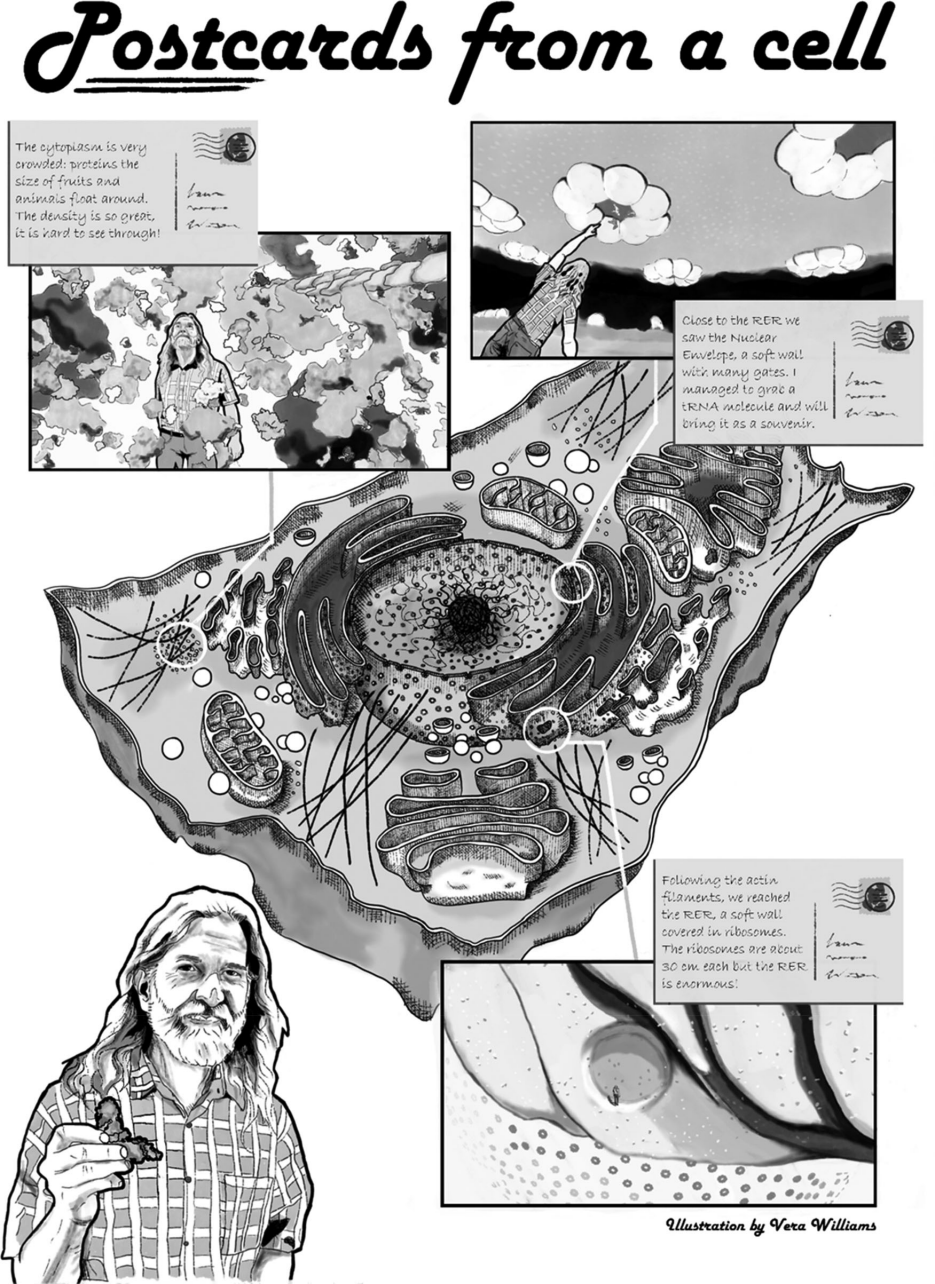
An artistic interpretation of what it would be like to visit a eukaryotic cell on the Ten Million Times scale. On this scale we can relate molecular objects to our everyday experience. There are molecules the size of fruits and animals, cytoskeletal elements resembling ropes, and a nucleus the size of a huge dome, just to name a few. To bring all these elements into perspective, we relate to David as an intuitive scale bar in this fictious world. Seeing molecular objects in relation to human size can help us understand their smallness or enormity with our instinctual understanding of size differences. PDB codes used as references: 5KY6, 3PGM, 4B4T, 1J5E, 1ASY, 2W4U, and 1EHZ.

Figure 3 reports a tribute to David Goodsell, in the form of Postcards from an Imaginary Trip to a cell. A fully verbal description is in the Supporting Information.

However, beside images, we also have a large amount of information from different sources that provide valuable inputs for our imagination. For example: lysosome acidification due to the activity of the proton pump; or actin/myosin contraction activated by a cascade involving electrical signals and Calcium waves. The exercise of imagining cells like towns, having accepted the scale resizing, implies an effort to translate these pieces of information into something that we can relate to and recognize once we learn to interpret them.

In this case our effort requires a step beyond the operations of Vesalius or Ramon y Cajal, as we have no access to visual nor to perceptual knowledge of pH, Calcium waves, or H‐bond formation. In the process of imaginary building the cell alive, we would like to be able to mentally figure much more than postcards.

## 4 A POSSIBLE WAY FORWARD

The imagination exercise easily creates different images in every reader, depending on our personal tastes and on our background knowledge: our effort should now be to merge them into a consistent common imagery. This common imagery could be the basis for another imaginary exercise: the planning for a full VR experience, in which we can “play” in a VR‐cell like we play in the VR representing our real world: feel it as if we were living it.

For example, in the case of our simple trip, we face the problem of how to visually characterize the difference (beyond shape) between a generic “protein surface” and a generic “Nucleic Acid surface,” so that they can be spotted from afar, like we can characterize wood from cloth or stone, even when details cannot be distinguished.

Table 3 reports a list of components that can be represented. The list is elaborated from a table prepared during two meetings dedicated to identifying challenges in structural and cellular visualization, with the contribution of scientists from different disciplines, including semiotics, communication design, color theory in addition to biology (Humphrey et al., 1996). Each entry presents its specific challenge, including, for example, the selection of textures and/or graphic effects that may be applied to make them visible/perceivable.

**TABLE 3:** Challenges of representation (non‐technical).

Small objects	Atoms and other small molecules	Known
Atoms to proteins	H_2_O	Known (but problematic)
	Carbohydrates (single)	Known
	Carbohydrates (chains)	Variable
	Lipids (layer)	Known
	Lipids (single)	Known
	Proteins and complexes	Reasonably known
	Nuclei acids	Known at small scale
Large objects	Organelles	Variable, but known
	Macromolecular complexes	Reasonably known
	Complex components (ribosomes, virus)	Known
Chem‐Phys features	Van der Waals	Chemical/physical
	H‐bonds	Chemical/physical
	Electric charges	Chemical/physical
	pH	Environment (chemical/physical)
	RedOx	Environment (chemical/physical)
	Temperature	Environment (physical)
Other	Activities	
	Allosteric effects	
	Flexibility/disorder	
	Uncertainty	
	Un‐knowns	
	Time/speed	
	Visitor (agent)	

*Note*: In the first block are listed objects whose shapes are known: in these cases, the challenge relates to the choice of features that allow viewers to detect their “material” and activities. The second block presents different challenges, as their representation should allow recognition of physical features that we can define, calculate, or measure, but have no visual correspondence in our experience. The third block reports an even more difficult list of items: we have no shared view of how we could report them to viewers.

To summarize the many challenges: how can we encode in visual terms concepts such as pH, RedOx, electric and lipophilic potentials? For example: could we imagine changes in pH like changes in temperature: colder more acidic, warmer more basic (or vice versa)? Or could we consider that a strongly reducing environment is a darker place, relative to an oxidizing one? And could a calcium wave be imagined as a sandstorm?

The options offered by special effects of computer graphics are many, and the selection of colors is only one of them: lights and shadows (source, color, intensity, direction, etc.), particles (size, motion, etc.), camera movements, all could be associated with various cellular situations, including the emotional effect of sound and music, and even spoken words. In addition, we will also have to find a suitable way to encode time.

**FIGURE 4:**
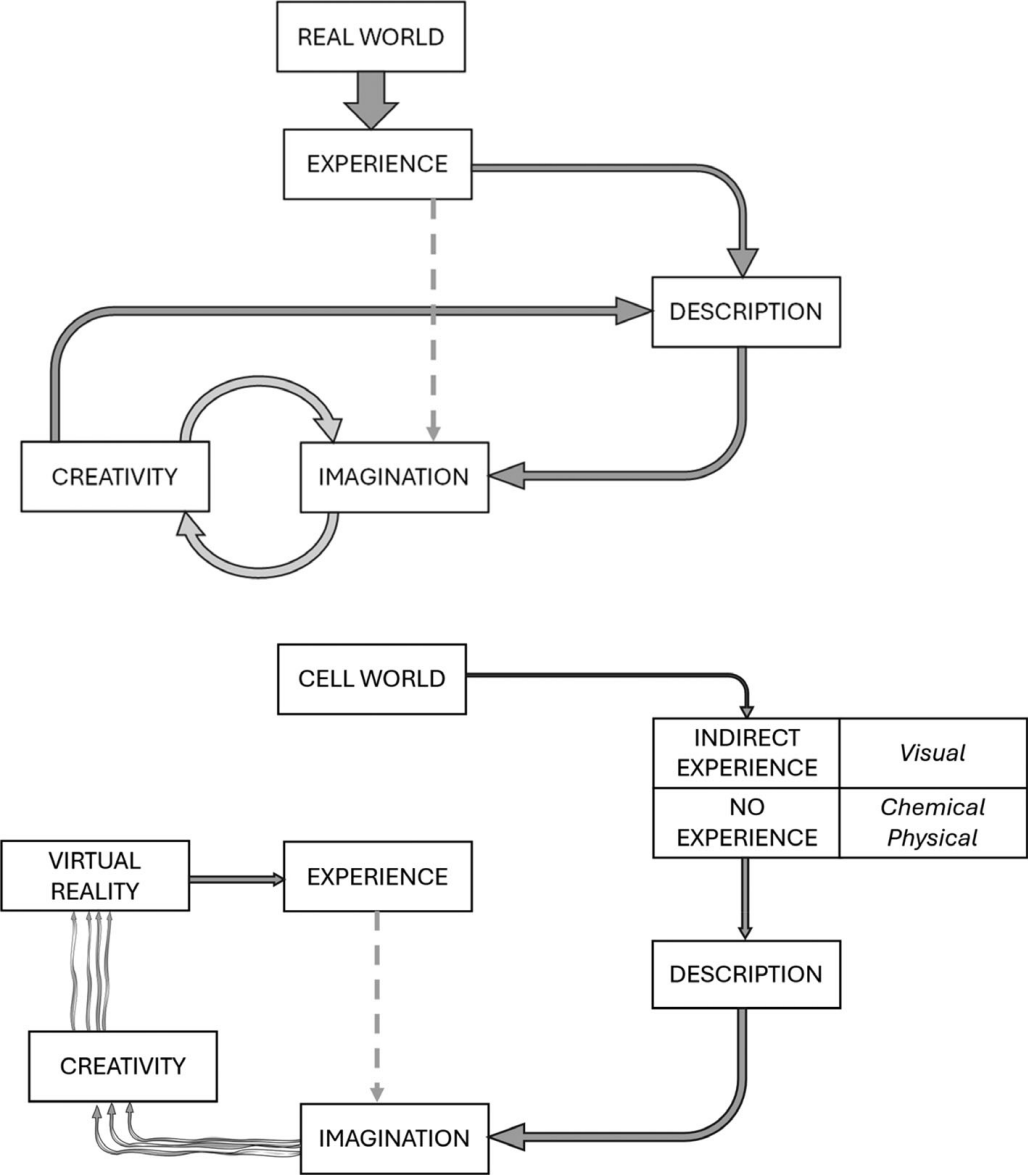
A schematic comparison of the different situations for describing the real world (top) versus the cellular world (bottom). The major direct link between reality and experience is missing in the case of cells, where our knowledge is derived from microscopy, mesoscale modeling, and structural biology, or obtained through experiments and other sophisticated techniques. These allow a reasonable description, such that we can imagine life in the cell, just like we can imagine described episodes from literature and other reports. Without the inputs from direct experience, if we aim at a more accessible understanding of the cellular world, we have to devise new, creative ways to describe (in VR or through other tools) the cellular phenomena that we know. These can be different for different individuals, here represented by a few wiggly lines.

The technology for making this dream a reality is not quite available yet, but might be not too far away. In my opinion, however, it would be premature to discuss technical issues at this point: as mentioned above, much progress has been made, with remarkable results. Yet, without a shared view of what it would feel like if we could really experience it, it will be hard to produce representations that can be easily recognized and interpreted by those who have never approached cellular biology. We do not yet know how to achieve such “shared imagination.” As shown in Figure 4, we could test several possibilities, or we could start an open discussion to collectively examine various options. However in order to let people build a mental imagery, it is important to have consistent representations, even in different styles, from comics to feature film or VR. The collective exercise here described might well contribute to a “standardized” visual definition of cells, of their behavior, and might finally lead to representing cells in a “photorealistic” way. There is a danger with photorealism, in that it may induce viewers into believing that what they see is actually real. The issue deserves a discussion that cannot be resolved in this article. Here we simply perform an exercise: we *imagine* it as “real,” so that we can *represent* it in many ways. My point is that if we manage to imagine the cell in a similar way (i.e., if we develop our imagination effort in a shared, collective way) then our representations will be consistent, avoiding some confusion in viewers, and possibly introducing aspects that have not been represented yet.

Cellular trips offer infinite possible adventures: it is up to us to make them intelligible to all viewers.

Figure 4 reports a schematic abstract representation of the theoretical approach.

## 5 DAVID'S LEGACY

David Goodsell demonstrated that combining knowledge with imagination and creativity (for example in the use of colors) is the first step necessary to represent proteins, their activity, and their relationships in cells.

He is also graced with the gift of artistic talent, and has been able to convey much of the (imagined) beauty of the world beyond the visible. For those of us that do not share his rare talent, there are modern, computer‐aided graphics techniques that can help.

It is important to proceed cautiously, because the representations we develop in this time, when the fields of microscopies and of structural biology are getting close enough to fill the gap “in between” (the mesosphere), will likely influence the collective imagination of the cellular world.

This is an implicit call to David to please stay around and provide help and assistance in the exercise of forging a shared view of a world that we can imagine beautiful, very lively and interesting just like any other complex landscape.

## Supporting information


**Data S1.** Supporting Information.

## Data Availability

Data sharing not applicable to this article as no datasets were generated or analyzed during the current study.

## References

[pro70616-bib-0001] Berry D . WEHI TV. n.d. [cited May 5, 2026]. Available from: http://www.wehi.edu.au/wehi-tv/wehitv

[pro70616-bib-0002] Chareshneu A , Midlik A , Ionescu C‐M , Rose A , Horský V , Cantara A , et al. Mol* volumes and segmentations: visualization and interpretation of cell imaging data alongside macromolecular structure data and biological annotations. Nucleic Acids Res. 2023;51:W326–W330. 37194693 10.1093/nar/gkad411 PMC10320116

[pro70616-bib-0003] Chen M . Rendering protein structures inside cells at the atomic level with unreal engine. Sci Commun Educ. 2023. 10.1101/2023.12.08.570879

[pro70616-bib-0004] Fiorentini E . Inducing visibilities: an attempt at Santiago Ramón y Cajal's aesthetic epistemology. Stud Hist Philos Biol Biomed Sci. 2011;42:391–394. 22035711 10.1016/j.shpsc.2011.07.008

[pro70616-bib-0005] Goodsell DS , Autin L , Olson AJ . Illustrate: software for biomolecular illustration. Structure. 2019;27:1716–1720.e1. 31519398 10.1016/j.str.2019.08.011 PMC6834899

[pro70616-bib-0006] GRC . Visualization in science and education conference. 2007 [cited May 5, 2026 ]. Available from: https://www.grc.org/visualization-in-science-and-education-conference/2007/

[pro70616-bib-0007] Humphrey W , Dalke A , Schulten K . VMD: visual molecular dynamics. J Mol Graph. 1996;14:33–38, 27–8. 8744570 10.1016/0263-7855(96)00018-5

[pro70616-bib-0009] Johnson GT , Autin L , Al‐Alusi M , Goodsell DS , Sanner MF , Olson AJ . cellPACK: a virtual mesoscope to model and visualize structural systems biology. Nat Methods. 2015;12:85–91. 10.1038/3204 25437435 PMC4281296

[pro70616-bib-0010] Johnston B , Maradani P , Elferich J , Davidson RB , Zhuang Y , Yao Y , et al. BradyAJohnston/MolecularNodes: V4.5.5. Zenodo, released November 9. 2025. https://zenodo.org/records/17368878

[pro70616-bib-0011] Maritan M , Autin L , Karr J , Covert MW , Olson AJ , Goodsell DS . Building structural models of a whole mycoplasma cell. J Mol Biol. 2022;434:167351. 34774566 10.1016/j.jmb.2021.167351 PMC8752489

[pro70616-bib-0013] Meowtabolism—examining feline behavior within unicellular algae. n.d. [cited may 5, 2026]. Available from: https://store.steampowered.com/app/4045010/Meowtabolism__Examining_Feline_Behavior_within_Unicellular_Algae/

[pro70616-bib-0012] SciVis . Scientific Visualization Unit SciVis Videos. n.d. [cited May 6, 2026]. Available from: http://www.scivis.it/videos

[pro70616-bib-0008] Steinegger J . The logistics of life. n.d. [cited may 5, 2026]. Available from: https://www.johannsteinegger.com/fungalcell

[pro70616-bib-0014] XVIVO . Medical animation—3D animation company. n.d. [cited May 5, 2026]. Available from: https://xvivo.com/

[pro70616-bib-0015] Zardecki C , Dutta S , Goodsell DS , Lowe R , Voigt M , Burley SK . pdb‐101: educational resources supporting molecular explorations through biology and medicine. Protein Sci. 2022;31:129–140. 34601771 10.1002/pro.4200 PMC8740840

[pro70616-bib-0016] Zoppè M . Towards a perceptive understanding of size in cellular biology. Nat Methods. 2017;14:662–665. 10.1038/nmeth.4300 28661495

[pro70616-bib-0017] Zoppè M . Colors in the representation of biological structures. J Integr Bioinform. 2022. 10.1515/jib-2022-0021 PMC9377705 35786236

