# Supplementary material for: Cellular and molecular representations: Following the lesson of David Goodsell
Source: Protein Sci. 2026 May 25;35(6):e70616. doi: 10.1002/pro.70616 (PMC13239077; doi:10.1002/pro.70616)
Supplement: Supplementary file 1 — Data S1. Supporting Information. [file PRO-35-e70616-s001.pdf]

Tourists in a cell

In this trip, we visit a completely new location, as if it were on another planet, with different places, different 'plants', 'animals' and 'machines', different activities going on… The first thing we do, in such place, is to take a look around, just like we would do if we visited a town never seen before.

The cell we visit is enlarged by ten million times, and the trip starts with a scroll through the most important places to take a general look: just like in a new town we reach the main square, with the temple/cathedral and the town hall, we get acquainted with the city plan, its radial or squared development, we walk around, and finally buy some souvenirs to take home, visiting the cell we start form the centre, as if we reached it by train. We find ourselves in a dense medium, through which we can *dive,* something in between flying and swimming through a dense liquid, that we can easily breath. The place is very crowded: proteins and complexes float around, different shapes remind us of fruits, animals or human artifacts, but not quite. The amount and density of all these objects (mostly proteins and small molecules), is such that they impede sight beyond a few spans, like in a dense school of fish. For this reason, we notice large things, those that we can measure in meters, like boats, airplanes or buildings, only when we almost bump into them.

Among the many things around, we notice what looks like a thick, long rope, similar to those used by that large boats to dock the mooring lines.

It is an actin filament, and there are several of them running in a bundle, more or less parallel, with some of them diverging towards somewhere else. We grab the ropes, and follow the main bundle; after few hundred meters we reach an area, actually a volume, dominated by a large structure: we see a kind of soft wall, covered with decorations distributed in a spiralling shape.

It is the Rough Endoplasmic Reticulum (RER), and the 'decorations' are chains of ribosomes, complex objects of about 30 cm, connected by a thin filament that seems to shift from one ribosome to the next (the mRNA). The mRNA filament seems to be suspended over the soft surface into which the ribosomes are clumped; there is a stream of visiting objects, of smaller size, that seem to touch briefly and leave. We don't have the time to stop and observe the details, but our guide explains that the small L-shaped things are tRNAs that deliver single aminoacids to the ribosomes, and that the ribosomes use aminoacids to build proteins. We don't see the proteins because they are inserted directly into the RER.

We proceed towards the border of the RER, where the surface bends by 180° to form a narrow sac. Our guide explains that what we see is the cytoplasmic side of a double-layered membrane, just about 6 to 8 cm think, enclosing an environment with controlled internal conditions: the RedOx potential is -2, quite oxidizing relative to the cytoplasm where we are now (only mildly oxydizing).

Sounds interesting, and we may consider a future visit. As we keep going, we realize that there are many of these cisternae, and that they are connected with one another through channels, like corridors or tunnels that connects separate buildings, allowing direct communication among them.

This complex of structures extends for several hundred meters, and at some point we see the 'connecting corridor' leading to another structure, somehow similar, but much much larger, and with different *decorations* on the surface. These are roundish structures, kind of fat rings, about a meter wide, protruding towards the cytoplasm by half of their thickness, hard to define because of the intricate composition, and blurred by the activity of extrusions emanating from their surface. Once again, our guide provides explanations: we are facing the wall of the nucleus, the largest building in town (organelle in the cell), and the 'decorations' are the nuclear pore complexes. They are complex structures, made up of hundreds of different proteins, and what we see is only the cytoplasmic face. The pores extend through the entire thickness of the membranous structure, that, similarly to the ER seen before, is shaped like a sac, called *Nuclear envelope*, the structure that contains the DNA of the cell. We stop for a moment to look at the pores: the central hole is bustling with activity: there are flexible protein chains and peptides that obscure the sight through.

Only after some time we realize that they are gateways for entry/exit of different objects: mRNAs, tRNAs and ribosomal subunits are flowing out, while other proteins and materials go in: some apparently enter unchecked, wile others, especially large complexes, seem to require more complex procedures.

After following the surface of the nuclear envelope for a while, between ribosomes and pores, we reach yet another very curious structure, it reminds us of a bunch of thin columns; we wish to spend some time to admire it, but our guide invites us to take the fast route to another interesting place in the cell: along the way, we pass by few big membrane-bound structures, mitochondria, the Golgi, smaller vescicles; we get a glimpse of the complex architecture of the cytoskeleton with its different elements: pipes (the microtubules), bundles of ropes (actin filaments), and other filamentous structures. Suddenly our guide enthusiastically calls our attention to what seems to be a very rare sight: a strange object, as large as one of the many vescicles, but this one seems to be made only of proteins and RNA.

It is called Vault, and nobody knows what it is or does exactly, except that it is involved with some neural functions and also in cancerous cells. It looks like a sort of cage, reminds someone of the cages used to trap lobsters, other likens it to cases for dangerous chemicals; just less than a meter long, with a fat center, and an apparently rigid surface. We feel lucky to have seen one, even if only briefly, and we don't really know why.

Flowing on the wake of a big ball transported along a microtubule track, we reach the periphery of the cell. We stop here to rest, wrap up the day and plan for our future visits. We are just besides a bunch of thin columns, similar to those seen before, arranged in triplets around a 2 or 3 m circle. Our guide calls Basal Body: it is the cytoplasmic root of a long cellular protrusion, called Cilium. We can't really see it, but apparently the thin columns extend for several tens of meters, providing a skeleton for the cilium.
Before we leave, a fascinating phenomenon happens: we are close to the soft wall that in this case is the internal face of the plasma membrane, with its peculiar decorations; We hear a noise, and see the wall deform with a bulge towards us. Suddenly many long proteins gather to build a net around it, the bulge grows until it becomes a spherical vescicle, it detaches from the wall and moves to the cell interior. Our guide calls it Clathrin mediated endocytosis.

Our trip is concluded, and on the way back, many of us reveal to have collected just a few objects, to examine them in details once back at home. Knowledge of single proteins is invaluable for better understanding what we have seen (and it will help in building a more detailed mental image of what happens, and how). In the comfort of our laboratories, we can examine single proteins, complexes and other molecules: we can dissect them, not using scalpel and formalin, but a series of bioinformatics tools. We can check every atom, their position, their charge and/or their involvement in catalytic activity. We can explore specific sites in the protein, such as mutation spots and evolutionary constraints. It becomes easy to recognize the flexible and more rigid parts of the proteins, where they interact with other proteins or co-factors, and how they may move, by comparing different static models and dynamic simulations. Through vocal commands or using our fingers (haptics), we turn around things, peel them, and feel the presence of charges, H-bond and Van del Waals forces. Someone likens the feelings to snapping a stud, opening a zip, or a velcro, or getting magnets close one another,
In the case of complexes, separating subunits by hand (through haptics) let us feel the properties that make them stick together, a process that reveal its importance when we try to recombine subunits, in their original or different conformations.
